# Chemical Profile and Use of the Peat as an Adsorbent for Extraction of Volatile Compounds from Leaves of Geranium (*Pelargonium graveolens* L’ Herit)

**DOI:** 10.3390/molecules25214923

**Published:** 2020-10-24

**Authors:** Edenilson dos Santos Niculau, Péricles Barreto Alves, Paulo Cesar de Lima Nogueira, Luciane Pimenta Cruz Romão, Graziele da Costa Cunha, Arie Fitzgerald Blank, Anderson de Carvalho Silva

**Affiliations:** 1 Curso de Química, Centro de Ciências Integradas, Universidade Federal do Tocantins, Av. Paraguai, s/n–esquina com Rua Uxiramas, Araguaína 77824-838, TO, Brazil; 2 Departamento de Química, Universidade Federal de Sergipe, São Cristóvão 49100-000, SE, Brazil; periclesbalves@gmail.com (P.B.A.); pclimanog@uol.com.br (P.C.d.L.N.); lucianeromao@uol.com.br (L.P.C.R.); grazy.ufs@gmail.com (G.d.C.C.); 3 Departamento de Engenharia Agronômica, Universidade Federal de Sergipe, São Cristóvão 49100-000, Brazil; arie.blank@gmail.com (A.F.B.); andersoncs.bio@hotmail.com (A.d.C.S.)

**Keywords:** *Pelargonium graveolens*, peat, porapak Q, volatile organic compounds, GC/MS

## Abstract

Volatile organic compounds (VOCs) from leaves of geranium (*Pelargonium graveolens* L’ Herit) were extracted by dynamic headspace using Porapak Q (HSD-P) as adsorbent and peat, a novel adsorbent in the extraction of plant volatiles, analyzed by gas chromatography–mass spectrometry (GC/MS) and gas chromatography–flame ionization (GC/FID), and the results were compared with those obtained by hydrodistillation (HD). The yield volatiles changed with the extraction method. HD was more efficient for extracting linalool (11.19%) and citronellyl formate (9.41%). Citronellol (28.06%), geraniol (38.26%) and 6,9-guaiadiene (9.55%) and geranyl tiglate (8.21%) were the major components identified by dynamic headspace using peat (HSD-T), while citronellol (16.88%), geraniol (13.63%), 6,9-guaiadiene (16.98%) and citronellyl formate (6.95%) were identified by dynamic headspace using Porapak Q (HSD-P). Furthermore, this work showed, for the first time, that in natura peat is useful to extract VOCs from leaves of geranium.

## 1. Introduction

*Pelargonium* is one of the five genera of the Geraniaceae family and contains about 280 species that are particularly rich in volatile oils, perennial aromatic crops of South African origin. A number of *Pelargonium* species (i.e., *P. graveolens, P. capitatum, P. zonale, P. roseum, P. odoratissimum*, and related hybrids) provide a highly marketed rose-scented essential oil, rich in citronellol and geraniol, obtained by steam distillation from fresh leaves and branches. The essential oil is mainly known under the generic name of “Geranium oil” even if obtained from distinct *Pelargonium* species and/or hybrids [1,2].

*P. graveolens* L’ Herit is a variety that is very odorous. The essential oil, which contains geranial, (*Z)*-rose oxide, isomenthone and linalool, is widely used in pharmaceutical and perfumery industries, food preservation and for flavoring of several food products [3,4,5,6].

Separation of volatile aroma compounds from botanical samples is most often achieved by several conventional sample preparation methods such as steam distillation, solvent extraction, enfleurage, maceration, cold pressing, supercritical extraction, purge and trap, and solid phase extraction (SPE) [7].

The extraction of volatile compounds by dynamic headspace (HSD) has been widely employed in the investigation of volatile organic compounds (VOCs) from various matrices such as beverages, plants, foods, cuisine, perfumery, juices, fruits and pheromones [8,9,10,11,12,13,14,15].

In HSD analysis, the sample is confined in an entrainment chamber and a carrier gas is passed over the sample. The VOCs released by the sample are carried by the gas to a solid trap, usually a porous organic polymer adsorbent such as Porapak Q (ethylvinylbenzene-divinylbenzene), where the analytes are adsorbed and preconcentrated. Trapped VOCs can be eluted from the adsorbing matrix into glass vials with thermal desorption, pure solvents or mixtures of low-boiling-point organic solvents. The merits of techniques for sampling the headspace for recovery of volatile compounds associated with the aroma has been known for some time and become a preferred method for capturing volatile [7,8]. Volatiles extracted are identified by gas chromatography–mass spectrometry (GC/MS) and can be semiquantificated by gas chromatography–flame ionization (GC/FID) [8,9,10,11,12,13,14,15].

During recent years, research on new materials for extraction, purification and separation processes of organic compounds in a wide polarity range has also been proposed by the growing interest for environmental preservation and human health protection. In view of this, peat is a sedentarily accumulated material consisting of dead organic matter in the waterlogged environment. Due to their high content of humic substances, natural peat exhibits favorable physicochemical properties enabling the application in various technical areas, for instance wastewater treatment, pollution monitoring, fuel production, soil fertilizing, and veterinary and human medicine [16,17]. Peat, as an adsorbent, is a porous material which can adsorb large quantities of metals, dyes and other organic molecules, whose adsorption capacity is comparable to conventional extracting phase like activated carbon, silica or alumina [18,19]. According to Mohan and Pittman [20], the price of peat is USD 0.09 kg^−1^, while the commercial price of Porapak Q is USD 4.56 g^−1^ [21]. Thus, peat is more economically viable if used for both analytical and industrial purposes.

To our knowledge there are no reports on the extraction of volatile compounds from geranium by dynamic headspace using Porapak Q and in natura peat as adsorbents. Therefore, the aim of the research was determining the chemical composition of volatiles from fresh leaves of geranium (*P. graveolens*) by HSD-T and HSD-P as the adsorbent material and compare it with those obtained by hydrodistillation (HD).

## 2. Results and Discussion

Previous work [22] shows that peat from Santo Amaro das Brotas municipality, Sergipe state, northeastern Brazil, is rich in C (53.1%), H (6.0%), O (31.5%) and dry ash (9.4%). Typical compositions of peat are in the range 40–60% C and 4–6% H_2_O. The elemental ratios H/C (1.3) and O/C (0.4) are indicators for the percentage saturation of the C atoms within the organic molecule and of the carbohydrate content, respectively [22]. Lower H/C ratios indicate higher aromaticity in the samples. The lowest O/C ratio of the peat sample indicates the lowest carbohydrate level and/or the highest organic content of that peat sample. The estimated value of the organic matter was 96% for the peat sample [22].

Detailed examination of the mineralogy of peat sample using X-ray diffraction (XRD) shows that it is characteristic of amorphous matter with a hump between 18° and 32° [22]. Bozkurt et al. [23] analyzing the processes involved in peat formation, recognized an anaerobic thick structural layer, which is formed of residual material from the original plant structure, decay products and new substances produced mainly by bacteria. At this level, peat would be amorphous and highly humified. However, only the XRD of dry ash or residue of that sample revealed mineral characteristics with presence of quartz mineral and some clay material. The combination of the elemental analyses and XRD indicates that the peat studied is highly humified and rich in organic compounds.

**Figure 1:**
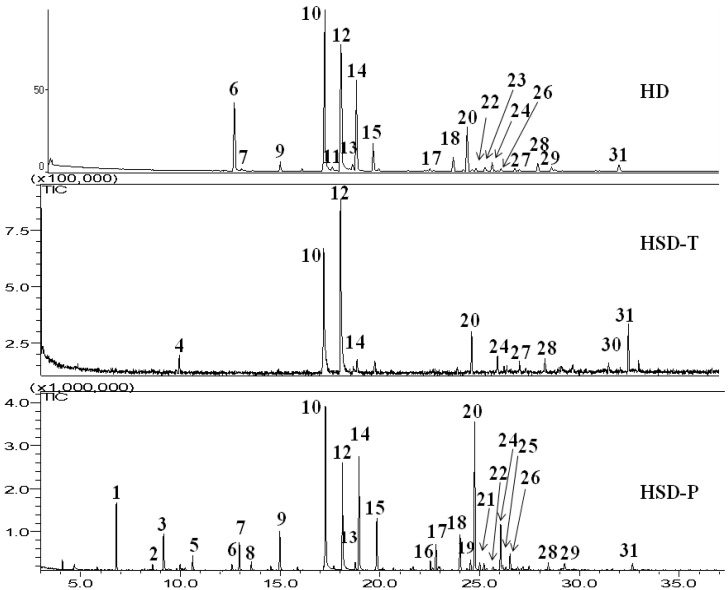
GC/MS chromatograms of volatile organic compounds (VOCs) *P. graveolens* using different sampling techniques: hydrodistillation (HD), dynamic headspace using Porapak Q (HSD-P) and peat as adsorbent (HSD-P). Number above each peak corresponding to the peak numbers in Table 1.

**Figure 2:**
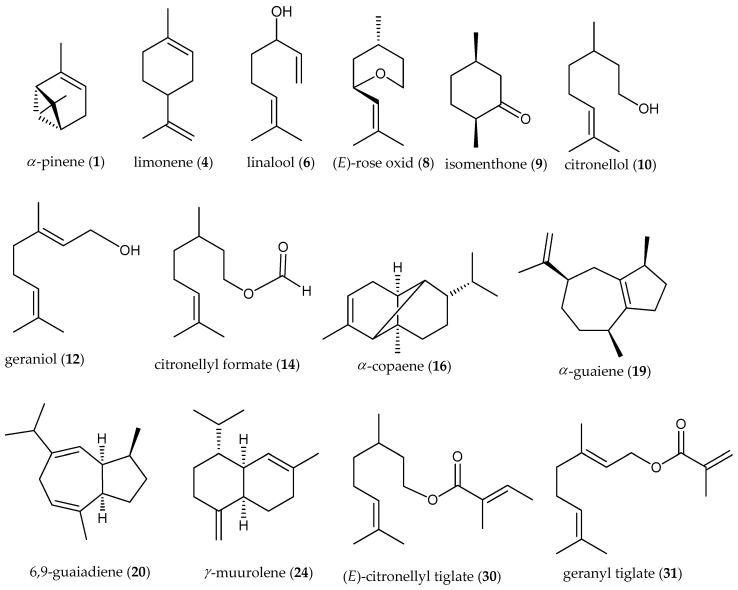
Main chemical compounds of *P. graveolens*.

VOCs were obtained by HD from fresh leaves of *P. graveolens*, which gave an oil in 0.15% yield based on fresh weight. Comparative total ion chromatogram (TIC) of the components identified at the three different extraction methods, HD, HSD-P and HSD-T, showed differences in volatile composition. Gas chromatography–flame ionization (GC/FID) normalized peak areas of the components identified are shown in Table 1. These VOCs can be classified into several chemical groups: monoterpenes hydrocarbons, oxygenated monoterpenes, sesquiterpenes hydrocarbons and esters. Oxygenated monoterpenes were present in the highest percentage in the three methods (65.50% in HD, 66.32% in HSD-T and 38.69% in HSD-P), while monoterpenes hydrocarbons were the minority (2.72% in HSD-T and 5.92% in HSD-P, respectively). Esters were the majority groups by HD and sesquiterpenes hydrocarbons (37.95%) by HSD-P. According to the literature, citronellol, geraniol, linalool and citronellyl formate are the main components present in the essential oil from fresh leaves of geranium obtained by HD [1,2,3,4,5]. This composition is consistent with the results of this work. Linalool (11.19%) and citronellyl formate (9.41%) had highest percentage by HD compared to HSD-T (not detected linalool and 1.92% citronellyl formate) and HSD-P (0.68% and 6.95%, respectively), Table 1, Figure 1 and Figure 2. The content of linalool in HD is statistically different between HSD-T and HSD-P, *p* < 0.05 and citronellyl formate only is statistically different in HSD-T (Table 1).

**Figure 3:**
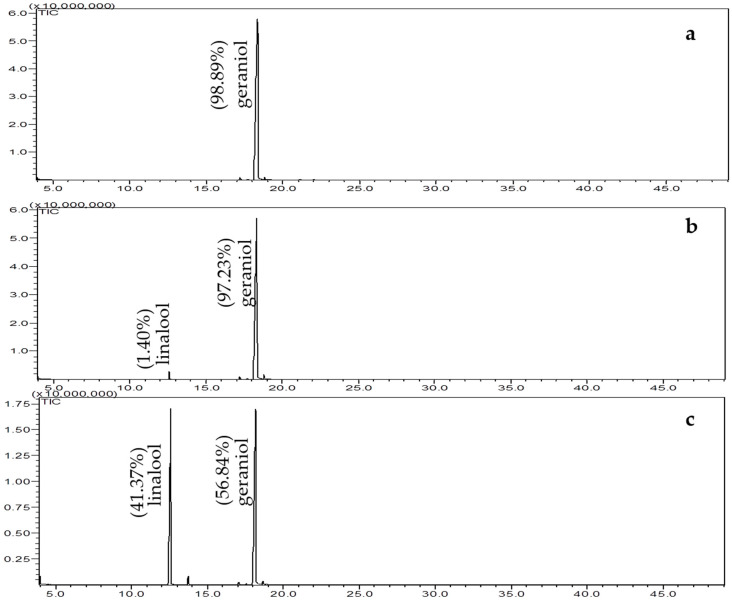
Chromatograms of the geraniol (**a**), hydrodistillation of geraniol (**b**) and solution of hydrodistillation of geraniol + linalool (**c**).

**Figure 4:**
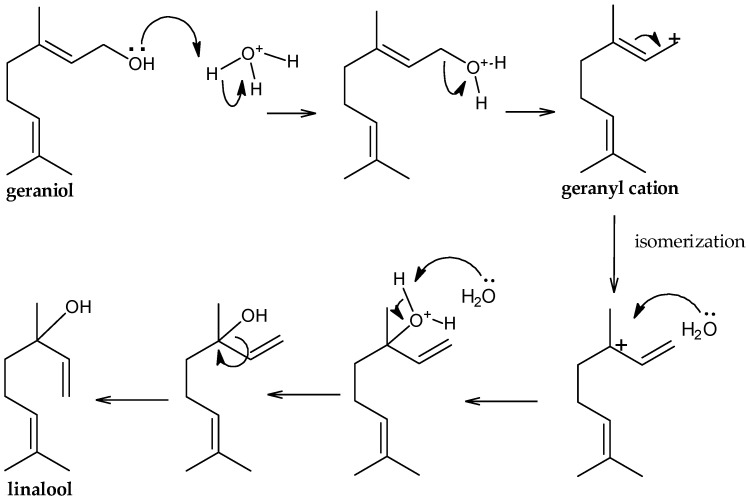
Proposed mechanism for conversion of geraniol to linalool under high temperature and water vapor.

Gomes et al. [24] applied the method of supercritical fluid extraction (EFS), a clean technology for the extraction of essential oil of geranium, and compared it with the extract obtained by HD, achieving low relative concentration of linalool by EFS in relation to the extraction by HD. The authors attributed this difference to linalool being formed through the geraniol when subjected to high temperatures in the presence of water vapor. This hypothesis was proven for the extraction methods used in this work, according Figure 3 and Figure 4. Proposed mechanism for conversion of geraniol to linalool is initiated by the capture of proton of the hydronium ion. Then, water is eliminated as a leaving group, generating the geranyl cation that isomerizes to form a tertiary carbocation. In the next steps, the formation of a C–O bond occurs by the nucleophilic attack of the water, deprotonation and rotation of the C–C sigma bond to form linalool (Figure 4).

Comparison of in natura pea as an adsorbent with Porapak Q available commercially showed that peat has a similar extracting power for *γ*-muurolene, and is better for geraniol, geranyl tiglate and limonene (*p* < 0.05). Dry ash, when used as an adsorbent, was unsatisfactory (data not shown). This fact shows that organic fraction is responsible for adsorption of the VOCs. In earlier work undertaken using the same peat that was employed in the present study, Romão et al. [22] demonstrated the presence of large quantities of aliphatic compounds. The interactions of organic compounds on material surfaces are not fully understood, it is generally acknowledged that the aromatic and aliphatic fractions of these adsorbents accumulate non-polar organic compounds, which are adsorbed by nonspecific attraction such as hydrophobic interactions [17].

Thus, HSD-T was more effective for extraction of geraniol (38.26%) and geranyl tiglate (8.21%) compared to extractions performed by HD (23.07% and 1.97%, respectively) and HSD-P (13.63% and 0.93%, respectively) according to statistical analysis, *p* < 0.05. Limonene (2.72%) and (*E*)-citronellyl tiglate (0.31%) were also identified by HSD-T and were not detected by HD and HSD-P (Table 1, Figure 1 and Figure 2). The content of limonene in HSD-T is statistically different between HD and HSD-P, *p* < 0.05.

Isomenthone (3.59%), 6,9-guaiadiene (16.98%) and *γ*-muurolene (6.64%) were identified in a higher percentage employing HSD-P in relation to HD (1.27%, 4.98% and 1.16%, respectively) and HSD-T (undetected isomenthone, 9.55% and 4.32%, respectively). *α*-Pinene (4.14%), (*E*)-rose oxide (0.71%), *α*-copaene (1.36%), and *α*-guaiene (1.44%) also identify by HSD-P, while these compounds were not detected by other methods (Table 1, Figure 1 and Figure 2). The contents of these compounds are statistically different between the HD and HSD-T, *p* < 0.05.

Citó et al. [15] also employed the technique of HSD-P and observed variations in the chemical composition of volatile fruit and leaves *Protium heptaphyllum*. Farag et al. [25] analyzed volatile compounds from the leaves of six species of *Juglans* and *Carya* by headspace dynamic using Tenax as adsorbent and hexane as eluting solvent and they found quantitative and qualitative differences among species. According to them, sesquiterpenes were the majority in all species studied and monoterpenes were detected at high levels in three from the six species under study, however, no work was performed using dynamic headspace and Porapak Q as an adsorbent for the extraction of volatile geranium. Peat also had never been used as an adsorbent for the extraction of plant volatiles.

## 3. Materials and Methods

### 3.1. Plant Material

*P. graveolens* was cultivated in the Research farm of the Federal University of Sergipe, Department of Agronomical Engineering, São Cristóvão municipality, Sergipe State, northeastern Brazil (10°56′ S, 37°05′ W), Brazil. Voucher specimens (ASE-14844) have been deposited in the Federal University of Sergipe Herbarium, CCBS, Biology Department, São Cristóvão, Sergipe, 49100-000, Brazil.

### 3.2. Collection, Preparation and Characterization of in Natura Peat and Dry Ash Material

In natura peat sample was collected from the depth 0–60 cm of the soil surface in Santo Amaro, Sergipe (S 10°48′56.2″; W 36°58′46.6″). Then it was stored in polyethylene bags to prevent action of light and humidity and conducted by the Laboratory of Environmental Analytical Chemistry/UFS. Subsequently, the sample was air dried for about three weeks in polyethylene trays previously decontaminated with nitric acid at 50%, and then the sample was crushed in porcelain mortar and passed through 115 mesh leggings.

Dry ash was also used for extraction of volatile and was obtained by burning peat in an oven at 750 °C for 4 h [22]. Characterization of peat was carried out according to previous work [22].

### 3.3. Isolation of the Volatile Compounds

#### 3.3.1. Hydrodistillation (HD)

Essential oil was obtained from the fresh leaves, by hydrodistillation in a Clevenger-type apparatus in triplicate until no more condensing oil could be seen (3 h) [7]. Samples were dried with anhydrous sodium sulfate and kept in amber vial at 4 °C until chromatographic analysis. Essential oil content (%) was calculated as the volume (mL) of essential oil per 100 g of fresh leave matter.

#### 3.3.2. Dynamic Headspace with Porapak Q as Adsorbent (HSD-P)

**Figure 5:**
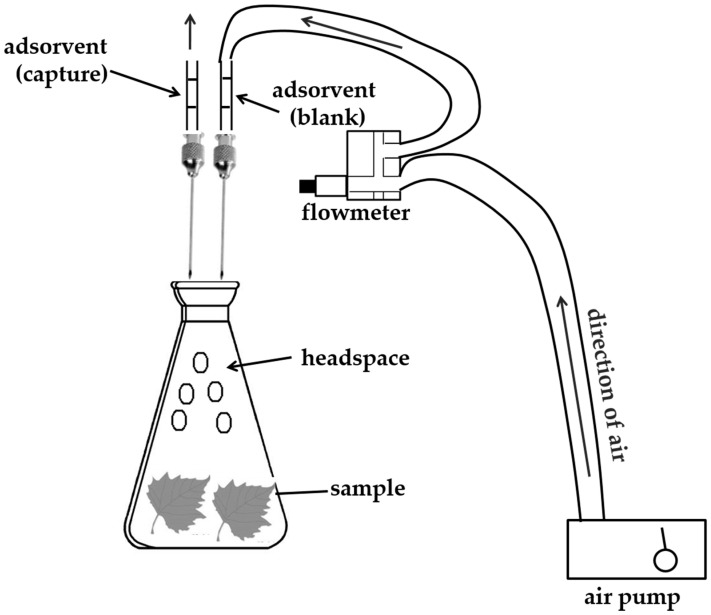
Scheme of volatile compounds sampling by dynamic headspace using Porapak Q (HSD-P) and peat as adsorbent (HSD-P).

Sixty grams (60 g) of fresh leaves were transferred to volumetric flask. The flask was connected to Porapak Q trap, which was connected in turn to air pump (Figure 5) [9]. The traps were prepared by packing Porapak Q (ca. 40 mg, 80–100 mesh Supelco) between silanized glass wool plugs in the glass tube which was packaged passing 6 mL MeOH (HPL grade) and 6 mL hexane (HPLC grade). A flow of air (ca. 0.5 L.min^−1^) was passed through the flasks containing the samples. Trapped volatiles were desorbed by solvent extraction (performed in triplicate) using hexane (1 mL, HPLC grade) by preliminary tests and applied a tone end of the column, and the solvent containing the eluted volatiles was forced to the other end of the column. The solvent volume was reduced carefully to approximately 200 µL in flow of nitrogen and an aliquot (1 µL) was immediately injected into the GC/MS and GC/FID.

The volume of solvent allowed easy manipulation and efficient elution without over-diluting the sample, for a trap dimension of 5 cm × 0.5 cm id. The best trapping period allowing detection of the greatest number of volatile constituents was found to be 3 h at room temperature.

#### 3.3.3. Dynamic Headspace with in Natura Peat as Adsorbent (HSD-T)

The relative extraction efficiencies with four preliminary solvents methylene chloride, methanol, ethyl acetate and n-hexane were investigated. The extraction of VOCs (performed in triplicate) by the technique of dynamic headspace were based on the solubility of peat in these solvents, and solutions were prepared separately in 80 mg of peat dissolved in 10 mL in the respective solvents. Only the n-hexane did not break the peat. Thus, this solvent was used for desorption of volatiles.

Sixty grams (60 g) of fresh leaves were transferred to volumetric flask. The flask was connected to peat trap, which was connected in turn to air pump (Figure 5) [9]. The traps were prepared by packing in natura peat (ca. 50 mg, 115 mesh) between silanized glass wool plugs in the glass tube which was packaged passing 6 mL n-hexane (HPLC grade). A flow of air (ca. 0.5 L.min^−1^) was passed through the flasks containing the samples. Trapped volatiles were desorbed by solvent extraction (performed in triplicate) using hexane (1 mL, HPLC grade) by preliminary tests and applied a tone end of the column, and the solvent containing the eluted volatiles was forced to the other end of the column. The solvent volume was reduced carefully to approximately 200 µL in flow of nitrogen and an aliquot (1 µL) was immediately injected into the GC/MS and GC/FID.

The size of the trap (5 cm × 0.5 cm i.d.), volume of solvent (hexane), temperature and time to capture the volatile were the same used for the dynamic headspace using Porapak Q.

### 3.4. Conversion of Geraniol to Linalool

One gram (1 g) of geraniol (98%, Sigma-Aldrich) was subjected to hydrodistillation in a Clevenger-type apparatus for 3 h using a 500 mL flask and approximately 250 mL of distilled water. After this time, hydrolate was subjected to extraction with ethyl acetate (3 × 50 mL) and the organic phase was dried with anhydrous Na_2_SO_4_, concentrated under reduced pressure in a rotary evaporator (Buchi, Flawil, Switzerland), transferred to an amber glass bottle, carefully concentrated in nitrogen and stored in freezer (Electrolux, Curitiba, Brazil) until analysis by GC/MS. To prove the conversion of geraniol to linalool, 7 mg of linalool (97%, Sigma-Aldrich, São Paulo, Brazil) was added to 3.33 mg/mL hydrolate solution (geraniol hydrodistillation solution, after conversion) and after GC/MS analysis was observed if peak of linalool increased. 

### 3.5. Gas Chromatography–Mass Spectrometry (GC/MS)

Essential oil analysis was performed on a Shimadzu QP5050A (Shimadzu Corporation, Kyoto, Japan) mass spectrometry system interfaced to gas chromatograph 17A instrument (GC/MS), employing the following conditions: column J&W Scientific DB-5MS (Folsom, CA, USA) fused silica capillary column (30 cm × 0.25 mm i.d. × 0.25 μm film thickness) composed of (5%-phenyl)-methylpolysiloxane, operating in electron impact mode at 70 eV; helium (99.999%) was used as carrier gas at a constant flow of 1.2 mL min^−1^ and an injection volume of 1.0 μL was employed (split ratio of 1:83) injector temperature 250 °C; interface temperature 280 °C. The oven temperature was programmed from 50 °C (isothermal for 2 min), with an increase of 4 °C/min, to 200 °C, then 10 °C/min to 300 °C, ending with a 10 min isothermal at 300 °C. Mass spectra were taken at 70 eV; a scan interval of 0.5 s and fragments from 40 to 550 Da.

### 3.6. Gas Chromatography–Flame Ionization (GC/FID)

Semiquantitative analysis of the chemical constituents was performed by flame ionization gas chromatography (FID), using a Shimadzu GC-17A (Shimadzu Corporation, Kyoto, Japan) equipment, under the following operational conditions: capillary ZB-5MS column (5% phenyl-arylene-95%-dimethylpolysiloxane) fused silica capillary column (30 m × 0.25 mm i.d. × 0.25 μm film thickness) from Phenomenex (Torrance, CA, USA), under the same conditions GC/MS. Quantification of each constituent was estimated by area normalization (%). Concentrations of compounds were calculated from the GC peak areas and they were arranged in order of GC elution.

### 3.7. Identification of Essential Oil Constituents

Identification of individual constituents was performed by computerized matching of the acquired mass spectra with those stored in NIST21 and NIST107 mass spectral library of the GC/MS data system. A linear hydrocarbon mixture of hydrocarbons (C_9_H_20_–C_19_H_40_) was injected under these same conditions and identification of constituents was then performed by comparing the spectra obtained with those of the equipment data bank and by retention indices (RI) for all compounds which were obtained with an equation proposed by Van den Dool and Kratz (1963) [26] for each constituent, as previously described [27].

### 3.8. Statistical Analysis

Statistical analysis was performed using Sisvar software, version 5.3. The data were subjected to variance analysis, one-way ANOVA, and the means of the percentage areas of each constituent were compared using the Tukey’s test (*p* < 0.05).

## 4. Conclusions

Volatile compounds from geranium (*P. graveolens*) changed according to the extraction method employed. The HD was more efficient in the extraction of linalool while geraniol, geranyl tiglate, limonene and (*E*)-citronellyl tiglate were identified in the highest percentage by HSD-T, and isomenthone, 6,9-guaiadiene by HSD-P.

The extraction carried out by dynamic headspace using dry ash as adsorbent was unsatisfactory, as no VOC was extracted, proving that the organic matter in peat is responsible for adsorption of volatile in the study. Therefore, in natura peat was employed in this work.

Identification and quantification of the volatile composition of *P. graveolens* by dynamic headspace, using peat as an adsorbent, were never carried out previously. Finally, considering that one of the objectives of this work was studying the scientific feasibility of alternative materials in the extraction of volatile plants, we emphasize that the peat can be used for the extraction of volatile compounds from leaves of geranium.

## Figures and Tables

**Table 1:** Composition of *P. graveolens* obtained by hydrodistillation (HD), Porapak Q (HSD-P) and peat (HSD-T).

N°	Compound Groups	RI ^#^	RI-lit ^##^	Content (%) **		
				HD	HSD-T	HSD-P
-	Monoterpenes hydrocarbons	-	-	0.00	2.72	5.92
1	*α*-pinene	931	932	0.00 *a	0.00 a	4.14 ± 2.00 b
2	myrcene	988	988	0.00 a	0.00 a	0.51 ± 0.02 b
4	limonene	1028	1024	0.00 a	2.72 ± 1.10 b	0.00 a
5	(*E*)-*β*-ocimene	1046	1044	0.00 a	0.00 a	1.27 ± 0.33 b
-	Oxygenated monoterpenes	-	-	65.50	66.32	38.69
6	linalool	1099	1095	11.19 ± 1.12 b	0.00 a	0.68 ± 0.12 a
7	(*Z*)-rose oxide	1109	1106	0.38 ± 0.05 b	0.00 a	2.47 ± 0.04 c
8	(*E*)-rose oxide	1126	1122	0.00 a	0.00 a	0.71 ± 0.07 b
9	isomenthone	1164	1158	1.27 ± 0.13 b	0.00 a	3.59 ± 0.52 c
10	citronellol	1226	1223	27.63 ± 0.48 a	28.06 ± 5.72 a	16.88 ± 0.02 a
11	neral	1238	1235	0.66 ± 0.06 b	0.00 a	0.00 a
12	geraniol	1250	1249	23.07 ± 1.63 b	38.26 ± 2.05 c	13.63 ± 1.35 a
13	geranial	1267	1264	1.30 ± 0.22 c	0.00 a	0.73 ± 0.03 b
-	Sesquiterpenes hydrocarbons	-	-	9.70	15.25	37.95
16	*α*-copaene	1373	1374	0.00 a	0.00 a	1.36 ± 0.19 b
17	*β*-borbonene	1382	1387	0.40 ± 0.04 b	0.00 a	2.76 ± 0.21 c
18	(*E*)-caryophyllene	1418	1417	1.70 ± 0.25 b	0.00 a	4.76 ± 0.38 c
19	*α*-guaiene	1434	1437	0.00 a	0.00 a	1.44 ± 0.02 b
20	6,9-guaiadiene	1439	1442	4.98 ± 0.62 a	9.55 ± 3.54 b	16.98 ± 0.40 c
21	*cis*-muurola-3,5-diene	1448	1448	0.00 a	0.00 a	0.95 ± 0.01 b
22	*α*−humulene	1454	1452	0.40 ± 0.06 a	0.00 a	0.49 ± 0.43 a
24	*γ*-muurolene	1479	1478	1.16 ± 0.24 a	4.32 ± 1.64 b	6.64 ± 0.32 b
25	germacrene D	1484	1484	0.00 a	0.00 a	0.10 ± 0.18 a
26	bicyclogermacrene	1493	1500	0.44 ± 0.08 a	0.00 a	2.47 ± 0.32 b
27	*δ*-amorphene	1516	1511	0.62 ± 0.15 a	1.38 ± 1.27 a	0.00a
-	Esters	-	-	18.77	13.74	15.24
3	(*3Z*)-hexenyl acetate	1005	1004	0.00 a	0.00 a	1.51 ± 0.20 b
14	citronellyl formate	1272	1271	9.41 ± 0.35 b	1.92 ± 1.77 a	6.95 ± 0.75 b
15	geranyl formate	1297	1298	3.92 ± 0.15 b	0.00 a	4.99 ± 0.29 c
23	linalool isovalerate	1468	1466	0.96 ± 0.03 b	0.00 a	0.00 a
28	geranyl butanoate	1554	1562	2.01 ± 0.12 a	3.30 ± 3.60 a	0.63 ± 0.55 a
29	2-phenyl ethyl tiglate	1582	1584	0.50 ± 0.45 a	0.00 a	0.23 ± 0.40 a
30	(*E*)- citronellyl tiglate	1662	1666	0.00 a	0.31 ± 0.54 a	0.00 a
31	geranyl tiglate	1694	1696	1.97 ± 0.11 a	8.21 ± 2.25 b	0.93 ± 0.03 a
	Total			93.97	98.03	97.80

Means followed by the same lowercase letter in the line do not differ between themselves by the Tukey test (*p* < 0.05). * Values are mean of three independent experiments. ** Percentages obtained by flame ionization (FID) peak area normalization. ^#^ Retention index relative to C9–C19 *n*-alkanes on the DB-5MS column. ^##^ Retention index from the literature Adams (2007).
